# Hepatitis C – Assessment to Treatment Trial (HepCATT) in primary care: study protocol for a cluster randomised controlled trial

**DOI:** 10.1186/s13063-016-1501-3

**Published:** 2016-07-29

**Authors:** Kirsty Roberts, John Macleod, Chris Metcalfe, Joanne Simon, Jeremy Horwood, William Hollingworth, Sharon Marlowe, Fiona H. Gordon, Peter Muir, Barbara Coleman, Peter Vickerman, Graham I. Harrison, Cherry-Ann Waldron, William Irving, Matthew Hickman

**Affiliations:** 1 School of Social and Community Medicine, University of Bristol, Bristol, UK; 2 Bristol Randomised Trials Collaboration, 39 Whatley Road, Bristol, UK; 3 University Hospitals Bristol, Bristol Royal Infirmary, Marlborough Street, Bristol, UK; 4 Public Health Laboratory Bristol, Public Health England, Myrtle Road, Bristol, UK; 5 Public Health Commissioning and Performance, Avonquay, Merchants Road, Cumberland Basin, Bristol, UK; 6 NIHR Nottingham Digestive Diseases Biomedical Research Unit, University Hospital Nottingham, Nottingham, UK; 7 South East Wales Trials Unit, Centre for Trials Research, Cardiff University, Cardiff, UK

**Keywords:** Hepatitis C, HCV, Case-finding, Complex intervention, Primary care, RCT

## Abstract

**Background:**

Public Health England (PHE) estimates that there are upwards of 160,000 individuals in England and Wales with chronic hepatitis C virus (HCV) infection, but until now only around 100,000 laboratory diagnoses have been reported to PHE and of these 28,000 have been treated. Targeted case-finding in primary care is estimated to be cost-effective; however, there has been no robust randomised controlled trial evidence available of specific interventions. Therefore, this study aims to develop and conduct a complex intervention within primary care and to evaluate this approach using a cluster randomised controlled trial.

**Methods/design:**

A total of 46 general practices in South West England will be randomised in a 1:1 ratio to receive either a complex intervention comprising: educational training on HCV for the practice; poster and leaflet display in the practice waiting rooms to raise awareness and encourage opportunistic testing; a HCV risk prediction algorithm based on information on possible risk markers in the electronic patient record run using Audit + software (BMJ Informatica). The audit will then be used to recall and offer patients a HCV test. Control practices will follow usual care. The effectiveness of the intervention will be measured by comparing number and rates of HCV testing, the number and proportion of patients testing positive, onward referral, rates of specialist assessment and treatment in control and intervention practices. Intervention costs and health service utilisation will be recorded to estimate the NHS cost per new HCV diagnosis and new HCV patient initiating treatment. Longer-term cost-effectiveness of the intervention in improving quality-adjusted life years (QALYs) will be extrapolated using a pre-existing dynamic health economic model. Patients’ and health care workers’ experiences and acceptability of the intervention will be explored through semi-structured qualitative interviews.

**Discussion:**

This trial has the potential to make an important impact on patient care and will provide high-quality evidence to help general practitioners make important decisions on HCV testing and onward referral. If found to be effective and cost-effective the intervention is readily scalable and can be used to support the implementation of NICE recommendations on HCV case-finding.

**Trial registration:**

ISRCTN61788850. Registered on 24 April 2015; Protocol Version: 2.0, 22 May 2015.

**Electronic supplementary material:**

The online version of this article (doi:10.1186/s13063-016-1501-3) contains supplementary material, which is available to authorized users.

## Background

Hepatitis C (HCV) infection is an important contributor to the growing burden of liver disease in the UK [1]. Infection results in cirrhosis and associated life-threatening complications in around 20 % of infected individuals over an average period of 20–30 years [2]. The risk factors for HCV infection include past or current injecting drug use, and being born or having lived in countries with a high prevalence of infection, where infection may have been acquired vertically, or through health care interventions. Approximately 90 % of infections acquired in the UK are among people who inject drugs (PWID) or who have injected drugs in the past [3]. Viral hepatitis, including HCV, is the only major cause of liver disease which is manageable or curable with recently developed therapies, as well as being preventable. HCV case-finding and uptake of treatment [4], however, need to be improved.

The sentinel surveillance of the Hepatitis Annual Report shows that general practice is the single most important setting for HCV testing (30 % of all tests originate here) and for identifying positive patients (31 % of all positive results are generated here). There is limited evidence from a small uncontrolled study in eight practices that targeted case-finding in primary care can increase the proportion of eligible individuals being tested for HCV by a factor of three and the proportion of positive tests amongst individuals tested by a factor of five [5]. The intervention involved offering practice staff training on the epidemiology, diagnosis and management of HCV infection, introducing an electronic patient record search to identify individuals at higher risk of HCV infection, and the offer of a HCV test to higher-risk individuals.

The intervention was used as the basis of a cost-effectiveness model of HCV case-finding in primary care used by the National Institute for Health and Care Excellence (NICE) [6]. Increasing testing in primary care (among those aged 30–54) was cost-effective at an estimated incremental cost-effectiveness ratio (ICER) of £13,900 per quality-adjusted life year (QALY) (79 to 93 % likely to be cost-effective at £20,000 and £30,000 per QALY gained willingness-to-pay thresholds). The intervention was more cost-effective if PWID who had an ongoing risk of transmitting HCV to other people were also tested and treated, and if the baseline testing rate was higher than expected. An additional potential benefit of this approach is that it may also identify patients who had already been diagnosed, but had not been referred for further management, offering an opportunity for reconsideration of such referrals [7].

In the US, a population-based approach has been adopted to identify HCV-infected individuals. Instead of risk-based testing, two individual models were evaluated based on membership of the 1945–1965 birth cohort or the presence of elevated levels of serum alanine aminotransferase (ALT) [8]. The birth cohort strategy was estimated to be cost-effective above a threshold HCV prevalence of 0.53 % which exceeds UK HCV prevalence and so is unlikely to be cost-effective in the UK [9]. ALT screening was previously investigated in a German study where primary care patients with an elevated ALT level of 50–100 IU/l had a HCV prevalence 10-fold above the population prevalence [10]. Since this study, the presence of one of the three significant risk factors for HCV (intravenous drug use, blood transfusion pre 1992 and immigration) or of elevated ALT levels has been shown to diagnose 83 % of unknown HCV-ribonucleic acid (RNA)-positive cases by screening only a quarter of the population studied [11]. The effectiveness of risk-based testing on its own in routine clinical practice is still under much debate. For example, individuals who have occasionally injected drugs in the remote past will probably not seek treatment for injecting drug use. Furthermore, they may be unlikely to disclose their risk status in a consultation with their general practitioner (GP). It has been estimated that from the total population of HCV-infected individuals in a high-income country, only 34 % are easily identifiable in high-risk groups such as a history of being injecting drug users or first-generation migrants [12]. Therefore, the hidden population of HCV-infected individuals may be significant and should be considered when developing future search strategies.

We aim to conduct a complex intervention study in primary care to identify patients who are at higher risk of HCV or patients who have a history of apparently untreated HCV and invite them to undergo testing. We will evaluate this approach using a cluster randomised controlled trial with qualitative and health economic components.

## Methods/design

### Study design

The study will consist of a pragmatic, two-arm, practice-level, cluster randomised controlled trial with a nested qualitative study and economic evaluation.

**Fig. 1:**
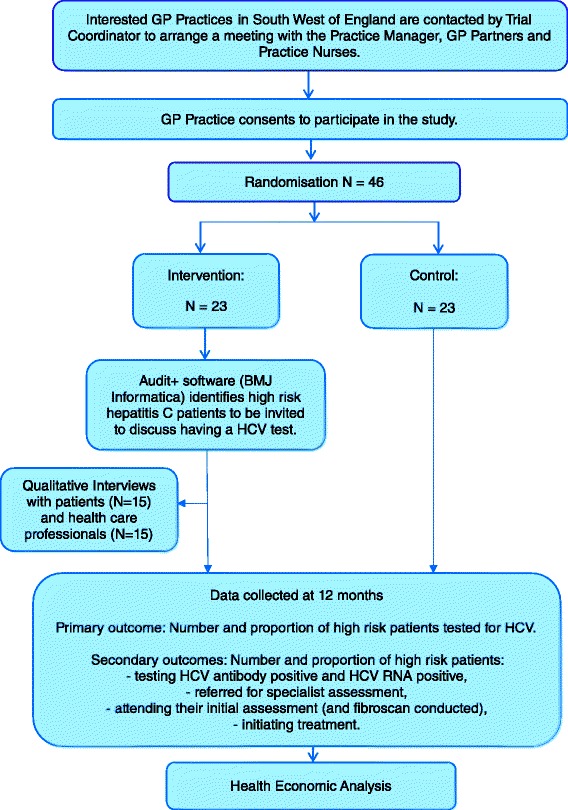
Trial schema and flow diagram

Ethics approval for the design described here was received from the National Research Ethics Service (NRES) Committee South West-Frenchay (Reference: 15/SW/0094) and research governance approvals obtained across all areas which will be involved in the study. The West of England National Institute for Health Research Clinical Research Network (NIHR CRN) will assist in identifying and accessing GP practices in the South West of England to participate in the trial. Interested practices will be contacted by the trial coordinator who will arrange a meeting with the practice manager, GP partners and practice nurse(s). Since this is a cluster randomised trial, and all patients within one practice will be treated in the same way, consent will be obtained from practices. Patients will not provide individual consent to participate in the intervention, though a purposeful sample will be asked for consent to participate in the nested qualitative study. See Fig. 1 for trial schema.

GP practices will be the unit of allocation. Practices will be randomised by an independent statistician in a 1:1 ratio to receive either the intervention or continue care as usual (control group). Randomisation will be stratified by area (Bristol, non-Bristol) and current rate of HCV testing (high: ≥1 % of the practice list versus low: <1 %), and minimised by practice size. In 93 practices in our target area, 15 (16 %) had HCV high testing rates.

### Participant eligibility

#### Qualitative study

Patients must be:

- Registered with one of the participating intervention practices and offered a HCV test
- Aged 18 years or over, able to provide consent and have access to a telephone

### Sample size

We based our initial sample size calculation on an average practice of 6500; with 4225 adults aged 15–65, we would expect approximately 1 % (42) of people to have an injecting history and in Bristol approximately 40 % (17) of those to have chronic HCV [13]. As a minimum we assume that 10 high-risk patients, based on the algorithm and markers of previous injecting history, will be identified and that the intervention increases uptake of HCV testing from 5 to 17 % of those high-risk patients. Then we require a sample size of 46 practices (23 intervention and 23 control, 230 high-risk patients identified in each arm) to achieve 90 % power at the 5 % significance level. We have assumed an intra-cluster correlation coefficient (ICC) of 0.05, and hence a design effect of 1.45, to accommodate variation in testing rates across practices.

This is a conservative sample size calculation to ensure a large enough number of practices and sufficient power to measure the main intervention effect on uptake of HCV case-finding and diagnosis as well as referral and assessment for treatment. First, practices being approached for the study and identified by the CRN have an average practice list size of 10,000 and, therefore, approximately 6500 adults aged 15–65. Second, it is likely that 2 % of adults will have an injecting history – combining estimates of people currently on opioid substitution treatment and ex-injecting drug users [14, 15]. Third, early pilot studies suggest that it is likely that 80 or more people for an average practice will be identified by the algorithm. This gives approximately 1840 people per arm and substantial power to detect a difference in uptake of HCV testing. In addition, if we assume that two thirds of people diagnosed with chronic HCV attend hepatology for assessment then the case-finding rates of 5 and 17 % convert to HCV treatment readiness rates of 1.3 and 4.4 % and 72 % power (with an ICC of 0.05) to detect a true difference in HCV treatment readiness. As we will adjust the statistical analysis for testing rate and whether a practice is within or outside the Bristol area, this will accommodate some of the clustering, and so it is likely that we will have sufficient power to detect a difference in HCV treatment assessment as well as HCV testing uptake.

### Planned interventions

#### Educational training on HCV

Participating practices will be offered a 1-hour educational presentation on HCV and trial procedures, including instructions on how to use the Audit + software (BMJ Informatica), which will be delivered by the trial coordinator at the practice. The practice will also be sent information about free online HCV educational resources (such as the RCGP e-learning module). All staff at the practice will be invited to attend the training session; however, if any members of staff cannot attend the pre-arranged date then the training can be cascaded within the practice.

#### Increasing patient awareness

Posters and leaflets will be displayed in the practice waiting room explaining risk factors for HCV infection. Practices will also be encouraged to include an additional question ‘have you ever injected drugs?’ to the new patient registration proforma. A positive answer would lead to discussion of testing at the next consultation.

#### Identification of patients with risk markers of HCV or patients previously diagnosed

The Audit + software (BMJ Informatica) will be used to assist practices in identifying patients with high-risk HCV markers or patients previously diagnosed who failed to initiate or complete treatment. Audit + is an integrated piece of software which is compatible with multiple GP clinical platforms and will have the HCV test algorithm or ‘audit’ built in. Audit + has been used in previous non-HCV-related studies and shown to reduce the number of patients ‘at risk and unscreened’ by almost two thirds [16]. We selected Audit + because it was becoming familiar to practices and was scalable.

The practice manager will be asked to install the Audit + software onto the practices’ clinical system. Practice staff training will include details on how to install and use the Audit + software as part of the intervention. Within the Audit + software, the HCV audit will be used to identify potentially eligible patients who have pre-defined Read codes defining them as ‘high-risk’ (see Appendix). The Audit + software identifies all patients with one or more of the codes. Patients excluded are those: tested recently for HCV; who have been referred to hepatology; receiving palliative care; who have raised ALT and tested for HCV. The audit will be run and updated every 24 hours on the GP system. GPs will be asked to screen the resultant list at the beginning of the intervention and then regularly, at intervals defined by the practice, to identify new patients during the remainder of the study period. GPs will be permitted to exclude any patient identified by the audit where they feel that an invitation for HCV testing or discussion of treatment is not appropriate. Eligible patients’ records identified by the audit will be automatically flagged by the software to encourage opportunistic testing if the patient attends the surgery. Practice staff can manually switch off the flagging should they feel that the patient is not eligible for a HCV test, if the patient declines a test or if the patient accepts the offer of a test, by entering a specific set of Read codes. Practice staff administrators will also be asked to send out a letter to each patient identified by the audit informing them that the practice is taking part in a study and that they may contact patients by telephone, e-mail or text (whichever is preferred by the practice) to book an appointment to discuss having a test. The patient invitation letters will be automatically processed using the integrated mail-merge facilities built within the software. Diagnosed patients are managed as usual/in standard care at the practice.

### Control practices

The control practices are only contacted at the time of randomisation and their contribution to the study (collection of data in 12 months’ time) is explained to them at the set-up telephone conversation shortly after they have been randomised. The study team do not contact the control practices again until at the end of the intervention period; control practices will be asked to install and run a ‘dummy version’ of the audit using the Audit + software to search for high-risk HCV patients retrospectively (i.e. the same calendar period as for the intervention practices). These data will be used to compare control and intervention practices to assess the impact of the intervention at the individual patient level as well as at the practice level. At the end of the study period, the control practices will be provided with the software licence for approximately 1 year and can choose to use any of its applications including the HCV audit.

### Outcome measures and statistical analysis

Data will be collected from three separate sources: general practices (using the Audit + software in the intervention practices and control practices), PHE laboratories Bristol and specialist secondary care hepatology services. During the study period we will also collect data on individuals who, from the practice electronic patient record (age, sex, postcode district, HCV risk factors as included in the algorithm) are identified by the audit as high-risk. As patient consent is not obtained, only anonymous data will be provided for the University of Bristol research team. Therefore, data will be sent by staff at the general practices and secondary care services to a data analyst at PHE laboratories. The data will then be linked using NHS numbers, GP code, name and date of birth, and anonymised and assigned a unique study ID number before sending the data to the research team at the University of Bristol.

#### Primary outcome measures

- *The number and proportion of high-risk patients tested for HCV*. The total number of HCV tests performed at the intervention practices and at the control practices during the intervention period will be collected by PHE laboratories

#### Secondary outcome measures

The HCV risk factors associated with each patient identified by the audit will be recorded by the Audit + software at participating practices. We will assess:

- *Number and proportion of high-risk patients testing HCV antibody positive and HCV RNA positive*. The results of each screening HCV test at each of the participating practices will be collected by PHE laboratories. Follow-up tests including polymerase chain reaction (PCR) (which diagnoses chronic HCV) and viral load results will also be collected by PHE laboratories
- *Number and proportion of high-risk patients referred for specialist assessment*. This will be recorded by data held by PHE laboratories for each of the participating practices
- *Number and proportion of high-risk patients attending their initial assessment* (*and fibroscan conducted*). This will be obtained from the secondary care hepatology records at University Hospitals Bristol and North Bristol Trusts and sent to PHE laboratories for linkage
- *Number and proportion of high-risk patients initiating treatment*. These data will be recorded by the secondary care hepatology records at University Hospitals Bristol and North Bristol Trusts and sent to PHE laboratories for linkage

The proportion of high-risk people tested for HCV, compared between intervention and control arms as a rate ratio, will be estimated in a negative binomial regression model, adjusted for whether an individual’s practice is in Bristol or not, and whether that practice has at baseline a high HCV testing rate or not. This regression model accommodates variation in the outcome measure between GP practices. This approach will be adapted to the secondary outcome measures, and also to estimate the risk difference between intervention and control arms for the primary outcome measure. These analyses will follow the intention-to-treat principle, with practices being analysed in the arms to which they are randomised.

### Health economics

We aim to estimate the short-term cost-effectiveness of the case-finding intervention from the NHS perspective. The research team will use a proforma to record the number of practices who accept the educational presentation on HCV, including the number of practice staff attending and job titles. Practice staff will be provided with a proforma to record the time taken to extract and screen the lists of high-risk patients and to exclude ineligible patients each time the Audit + search is run. The proforma will also ask practice staff to record the number of letters sent out to patients inviting them to book an appointment. At both intervention and control sites practice staff will extract information from the electronic patient record on the consultations, laboratory tests, radiology and prescriptions received by patients during the study period identified as high-risk by Audit +. This dataset will be de-identified and assigned a unique study ID by a data analyst at PHE laboratories, as described above.

This will allow us to identify HCV case-finding consultations (in the intervention practices) and opportunistic HCV testing consultations (in intervention and control practices). We will also use the linked dataset to identify post-diagnosis resource use (e.g. follow-up consultations with GPs and specialists, prescriptions). Where available, national unit costs [17, 18], or locally estimated unit costs if unavailable, will be used to value the time spent inviting patients to case-finding appointments and delivering HCV-related care to high-risk and untreated patients throughout the study period. We will calculate the incremental cost per new diagnosis of HCV and the incremental cost per new patient initiating HCV treatment in intervention versus control practices. Uncertainty will be explored using cost-effectiveness acceptability curves, which estimate the probability of intervention cost-effectiveness for various willingness-to-pay thresholds [19].

Provided that the intervention improves case-finding and treatment, we will extrapolate short-term costs and outcomes of the intervention using a health economic model that is parameterised to the UK health provider perspective including liver disease progression stages and QALYs used in previous exercises for NICE and the Human Tissue Authority (HTA) [7, 20–22]. The model also can be dynamic, incorporating the potential prevention benefit of diagnosing and treating people who inject drugs as well as people who have ceased injecting or other patient groups. Costs and health benefits are discounted at 3.5 % per year in the base case according to NICE guidelines. Results will be expressed in terms of incremental cost-effectiveness ratios (ICERs), calculated by dividing the difference in costs between the intervention and usual care strategies, divided by the difference in health outcomes, in this case QALYs. For each economic analysis, multivariate uncertainty analyses and numerous one-way sensitivity analyses will be undertaken so that the robustness of the model results can be examined; and we will also plot cost-effectiveness acceptability curves.

### Qualitative interviews

Health care professionals (HCPs) and patients’ views and experiences of the intervention will be examined through the conduct of semi-structured qualitative interviews. The qualitative findings will assist in highlighting the perceived effectiveness and acceptability of targeted HCV case-finding and increasing HCP and patient awareness within primary care.

#### Qualitative study design

Qualitative telephone interviews will be conducted with primary care patients and the interviews will consider and compare (1) views and experiences of being approached opportunistically at the practice or contacted by letter and invited for a consultation with their GP, (2) the acceptability of being approached for a HCV test, (3) views on whether any additional interventions may be helpful, and (4) (if tested HCV-positive) views and experiences of referral to secondary care and on-going assessment/treatment.

HCPs involved at the intervention practices will also be interviewed to gather data on (1) their views and experiences, (2) changes in clinical practice, (3) acceptability and feasibility of the intervention, (4) information and support needs, and (5) their attitudes to the future implementation of the intervention within primary care.

#### Interview participant sampling and recruitment

Patients offered a HCV test at participating intervention practices will be asked if they are willing to be contacted about taking part in a qualitative interview at the time of their consultation. From participants who indicate that they are willing to be contacted, a purposive sample will be drawn in relation to (1) primary care practice, and (2) socio-demographic variables (e.g. gender, age and ethnicity). Telephone interviews will be conducted with a sample of patients who (1) declined a HCV test, (2) accepted a HCV test, (3) tested positive for HCV and were subsequently referred to secondary care. HCP (including general practitioners and nurses) involved in implementing the intervention will be purposively sampled in relation to (1) primary care practice, (2) professional role, and (3) length of time since qualification/seniority.

Those sampled will be contacted by the researcher and asked if they are interested in taking part in an interview. Patients will be interviewed either after they have been offered a HCV test or for patients who have already tested positive for HCV before the study, a sample of these will be interviewed following their first attendance for assessment and/or treatment. The sample sizes will be determined by the need to achieve data saturation, such that no new themes are emerging from the data by the end of data collection [23]. This is likely to include up to 15 HCPs and 15 patients at participating intervention practices.

#### Interview conduct

All interviews will be conducted by telephone. Participants will be asked to provide their verbal informed consent to take part immediately before the interview. A flexible topic guide will be used in order to assist questioning during in-depth individual interviews. Topic guides will be modified as necessary throughout the course of the study to reflect findings as they emerge. With informed consent from participants, interviews will be audio-recorded using a digital voice recorder, transcribed and anonymised to protect confidentiality.

#### Qualitative data analysis

Interview transcripts will be checked for accuracy and then imported into NVivo10 qualitative data analysis software to aid management and analysis of data. Analysis will begin shortly after data collection starts and will be ongoing and iterative. Analysis will inform further data collection: for instance, analytic insights from data gathered in earlier interviews will help identify any changes that need to be made to the topic guide during later interviews. Thematic analysis [24], utilising a data-driven inductive approach will be used to scrutinise the data in order to identify and analyse patterns and themes of particular salience for participants and across the dataset.

## Discussion

This trial has the potential to make an important impact on patient care and will provide high-quality evidence to help general practitioners make important decisions on HCV testing and onward referral. The intervention is scalable and, therefore, if found to be effective and cost-effective can be readily rolled out and implemented elsewhere in the UK in response to new NICE recommendations on improving HCV case-finding.

## Trial status

At the time of manuscript submission, this trial was still in the practice recruitment phase.

## Abbreviations

ALT, alanine aminotransferase; CRN, Clinical Research Network; GP, general practitioner; HCP, health care professional; HCV, hepatitis C virus; HTA, Human Tissue Authority; ICC, intra-cluster correlation; ICER, incremental cost-effectiveness ratio; NHS, National Health Service; NICE, National Institute for Health and Care Excellence; NIHR, National Institute for Health Research; NRES, National Research Ethics Service; PCR, polymerase chain reaction; PHE, Public Health England; PWID, people who inject drugs; QALY, quality-adjusted life year; RCGP, Royal College of General Practitioners; RNA, ribonucleic acid

## Appendix

### HCV audit

A HCV test algorithm was developed and piloted at two local general practices before its integration into the Audit + software (BMJ Informatica). The HCV test algorithm was designed to identify registered patients with high-risk HCV markers or patients previously diagnosed who failed to initiate or complete treatment. The search terms included (1) age 18–75 years old, (2) previous intravenous drug use (IDU) history, (3) opiate prescription (including methadone or buprenorphine), (4) opiate misuse, (5) blood transfusion prior to 1991, (6) blood products prior to 1996, (7) transplant prior to 1992, (8) infection with HIV, (9) infection with hepatitis B virus (HBV), (10) born to a mother with HCV, (11) child in care, (12) prison history, (13) altered ALT levels, (14) patients tested for HCV over 1 year previous, before the intervention. Identified individuals considered by GPs to be unsuitable for a discussion about HCV testing or treatment were excluded. Patients also automatically excluded by the HCV audit included (1) patients referred to hepatology, (2) patients tested less than 1 year prior to the intervention, (3) patients receiving end of life/palliative care, and patients with raised ALT levels who have been tested for HCV.

The list of codes are available from the authors and given in Additional file 1. Spirit Checklist also available in Additional file 2.

## Additional file

Additional file 1: HepCATT study algorithm (Read codes). (DOC 118 kb) Additional file 2: SPIRIT 2013 Checklist: Recommended items to address in a clinical trial protocol and related documents. (DOC 121 kb)
