# Supplementary material for: Hepatitis C – Assessment to Treatment Trial (HepCATT) in primary care: study protocol for a cluster randomised controlled trial
Source: Trials. 2016 Jul 29;17:366. doi: 10.1186/s13063-016-1501-3 (PMC4966763; doi:10.1186/s13063-016-1501-3)
Supplement: Additional file 1: — HepCATT study algorithm (Read codes). (DOC 118 kb) [file 13063_2016_1501_MOESM1_ESM.doc]

**Web appendix HepCATT Study Algorithm (Read Codes)**

| Name | IDU | |
| --- | --- | --- |
| Description | History of Intravenous Drug use | |
| Selection | Latest | |
| Referenced by sections |  | |
| Referenced by measures | Call01, Off01, Tst01, Ref01, Refe01 | |
| Read V2 Terms | | CTV3 Terms |
| 13c0: Injecting drug user  13c1: Intravenous drug user  13c7: Current drug user  13cJ: Previously injecting drug user  146C: Failed heroin detoxification  1T0%: H/O heroin misuse  1TE: Uses heroin on top of substitution therapy  1TF: Does not use heroin on top of substitution therapy  E240: Opioid type drug dependence  Eu112: [X]Mental and behav dis due to use opioids: dependence syndr  SL501: Heroin poisoning  T800: Accidental poisoning by heroin  TJ50: Adverse reaction to heroin, diamorphine  U1A5: [X]Accident poisoning/exposure to narcotic drug  U205: [X]Intent self poison/exposure to narcotic drug  13c4: Intranasal drug user  1V3C: Shares needles  1V65: Heroin misuse  1V3M: Does not use needle and syringe exchange scheme  1V32: Neck injector  1V3B: Shares syringes  1V33: Groin injector  1V3G: Does not clean needles  1V35: Shares drug equipment  1V38: Sharing of drug injecting equipment  HMPNQDR1: "HMPNQDR1"  ZV115: [V]Personal history of drug abuse by injection  EMISNQCU1: "EMISNQCU1"  EMISNQPR6: "EMISNQPR6"  EMISNQND10: "EMISNQND10"  EMISNQHO4: "EMISNQHO4"  EMISNQMI10: "EMISNQMI10"  13c5: Substance misuse increased  13c6: Substance misuse decreased  13c8: Reduced drugs misuse  13c9: Subcutaneous drug user  13cC: Continuous use of drugs  13cD: Episodic use of drugs  13cF: Preoccupied with substance misuse  13cF: Preoccupied with substance misuse  13cH: Persistent substance misuse  13cM: Substance misuse  13cN: Has never shared drug injection equipment  146F: H/O: drug abuse  E248: Combined opioid with other drug dependence  8FB: Drug rehabilitation  8FB0: Drug detoxification programme completed  1283: FH: Drug dependency  1463: H/O: drug dependency  1J11: Suspected abuse hard drugs  1V0: Misuses drugs  1V3%: Drug injection behaviour  1V65: Heroin misuse  1P31: Compulsive drug taking | |  |

| Name | OPIATE_RX | |
| --- | --- | --- |
| Description | Methadone or buprenorphine prescriptions (excluding patches and tablets) | |
| Selection | Latest | |
| Referenced by sections |  | |
| Referenced by measures | Call02, Off02, Tst02, Ref02, Refe02 | |
| Read V2 Terms | | CTV3 Terms |
| djc%: METHADONE HCL [ANALGESIC]  dj32: TEMGESIC 300micrograms/1mL injection  dj33: TEMGESIC 600microgram/2mL injection  dj3y: BUPRENORPHINE 300microgram/1mL injection  dj3z: BUPRENORPHINE 600micrograms/2mL injection  dj3D: BUPRENORPHINE+NALOXONE 2mg/0.5mg sublingual tablets  dj3E: SUBOXONE 2mg/0.5mg sublingual tablets  dj3F: BUPRENORPHINE+NALOXONE 8mg/2mg sublingual tablets  dj3G: SUBOXONE 8mg/2mg sublingual tablets  djcA: METHADONE DILUENT liquid  cg51: METHADONE 2mg/5mL linctus | |  |

| Name | OPIATE_MISUSE | |
| --- | --- | --- |
| Description | Opiate misuse | |
| Selection | Latest | |
| Referenced by sections |  | |
| Referenced by measures | Call02, Off02, Tst02, Ref02, Refe02 | |
| Read V2 Terms | | CTV3 Terms |
| 13cG0: Opioid tolerant  13cG1: Opioid naive  4I71: Oral fluid opiate level  1T1%: H/O methadone misuse  44u1: Serum methadone level  44uK: Plasma methadone level  46QB: Urine methadone  46Qf: Urine methadone metabolite level  4I75: Oral fluid methadone level  8B23: Drug addiction therapy  8B2N: Drug addiction detoxification therapy - methadone  8B2P: Drug addiction maintenance therapy - methadone  8BE0: Reinduction to methadone maintenance therapy  SL502: Methadone poisoning  T801: Accidental poisoning by methadone  TJ51: Adverse reaction to methadone  U6050: [X]Opioids + relat analgesics caus advers eff in therap use  R10B4: [D]Finding of opiate drug in blood  679j0: Education about taking methadone | |  |

| Name | BLOOD_1991 | |
| --- | --- | --- |
| Description | Blood transfusion prior to 1991 | |
| Selection | Latest before 1 Jan 1991 | |
| Referenced by sections |  | |
| Referenced by measures | Call03, Off03, Tst03, Ref03, Refe03 | |
| Read V2 Terms | | CTV3 Terms |
| 14S1: H/O: blood transfusion  435: Transfusion centre ref. no.  7K1Q2: Transfusion of stem cells  7L13: Exchange blood transfusion  7L13y: Other specified exchange blood transfusion  7L13z: Exchange blood transfusion NOS  7L14-7L143: Other blood transfusion ... Intravenous blood transfusion NEC  7L14y: Other specified other blood transfusion  7L14z: Other blood transfusion NOS  7L15%: Other intravenous transfusion  !7L156: (Excluding) Plasmapharesis  88: Cardiovascular procedures  9bC1: Blood transfusion (specialty)  SP33: Infection after injection/infusion/transfusion/vaccination  SP332: Infection after transfusion  SP33z: Infection after injection/infusion/transfusion/vacc NOS  SP38: Other transfusion reaction  SP380: Septic shock due to transfusion  SP38z: Transfusion reaction NOS  TA30: Excess blood or other fluid during transfusion or infusion  TA41: Mechanical failure of apparatus during infusion/transfusion  TA411: Mechanical failure of apparatus during transfusion  TA41z: Mechanical failure of apparatus - infusion/transfusion NOS  TB1y0: Blood transfusion with complication, without blame  ZV582: [V]Blood transfusion, without reported diagnosis  ZVu3M: [X]Blood transfusion, without reported diagnosis  G8y00: Extravasation following blood transfusion  TJ47z: Adverse reaction to blood or blood products NOS  ZVu3V: [X]Blood transfusion | |  |

| Name | PRODUCT_1986 | |
| --- | --- | --- |
| Description | Blood products before 1986 | |
| Selection | Latest before 1 Jan 1986 | |
| Referenced by sections |  | |
| Referenced by measures | Call04, Off04, Tst04, Ref04, Refe04 | |
| Read V2 Terms | | CTV3 Terms |
| 7L141: Intravenous blood transfusion of packed cells  7L142: Intravenous blood transfusion of platelets  7L154: Transfusion of platelets NEC  7L150: Transfusion of coagulation factor  7L151: Transfusion of plasma  7L158: Transfusion of plasma NEC  7L152: Transfusion of serum NEC  TJ470: Adverse reaction to blood plasma  TJ471: Adverse reaction to human fibrinogen  TJ472: Adverse reaction to packed red cells | |  |

| Name | TRANSP_1992 | |
| --- | --- | --- |
| Description | Transplant before 1992 | |
| Selection | Latest | |
| Referenced by sections |  | |
| Referenced by measures | Call05, Off05, Tst05, Ref05, Refe05 | |
| Read V2 Terms | | CTV3 Terms |
| 8HBB: Transplant follow-up  7B015: Transplant nephrectomy  9b8K: Transplantation surgery  7450: Transplantation of lung  7800: Transplantation of liver  764C: Transplantation of ileum  7B00: Transplantation of kidney  78420: Transplantation of spleen  7830: Transplantation of pancreas  7901: Other transplantation of heart  9b8B2: Cardiothoracic transplantation  SP080: Transplanted organ failure  SP081: Transplanted organ rejection  SP083-SP086: Kidney transplant failure and rejection ... Liver transplant failure and rejection  SP089: Complication of transplanted lung  SP08C-SP08H: Accelerated rejection of renal transplant ... Acute rejection of renal transplant  SP08Z: Thrombosis of artery of transplanted kidney  ZV420: [V]Kidney transplanted  ZV421: [V]Heart transplanted  ZV426: [V]Lung transplanted  ZV427: [V]Liver transplanted  7B063: Exploration of renal transplant  7900: Transplantation of heart and lung  HNG0111: "HNG0111"  78052: Exploration of liver  8HkP: Referral to surgical transplant service | |  |

| Name | HIV | |
| --- | --- | --- |
| Description | Infection with HIV | |
| Selection | Latest | |
| Referenced by sections |  | |
| Referenced by measures | Call06, Off06, Tst06, Ref06, Refe06 | |
| Read V2 Terms | | CTV3 Terms |
| 43C3: HTLV-3 antibody positive  4J34: HIV viral load  A789: Human immunodef virus resulting in other disease  A788: Acquired immune deficiency syndrome  66j: Human immunodeficiency virus monitoring  Eu024: [X]Dementia in human immunodef virus [HIV] disease  4J3F: Human immunodeficiency virus viral load by log rank  L179: HIV disease complicating pregnancy childbirth puerperium  R109: [D]Laboratory evidence of human immunodeficiency virus [HIV]  43h9: HIV proviral deoxyribonucleic acid polymerase chain reaction  ZV01A: [V]Asymptomatic human immunodeficency virus infection status  9kl: HIV pos gen health check serv declind - enhanc service admin  EGTON41: "EGTON41"  EMISNQHO13: "EMISNQHO13"  AyuC: [X]Human immunodeficiency virus disease  HNG0143: "HNG0143"  HNG0607: "HNG0607"  43j7: HIV 1 nucleic acid detection  A788: Acquired immune deficiency syndrome  66j%: Human immunodeficiency virus monitoring  A789: Human immunodef virus resulting in other disease | |  |

| Name | HEP_B | |
| --- | --- | --- |
| Description | Infection with Hepatitis B | |
| Selection | Latest | |
| Referenced by sections |  | |
| Referenced by measures | Call07, Off07, Tst07, Ref07, Refe07 | |
| Read V2 Terms | | CTV3 Terms |
| 141E: History of hepatitis B  4J3D: Hepatitis B viral load  ZV02B: [V]Hepatitis B carrier  43B4: Hepatitis B surface antig +ve  7Q052: Hepatitis B treatment drugs Band 1  9kZ: Hepatitis B screening positive - enhanced services admin  A703: Viral (serum) hepatitis B  Q4091: Congenital hepatitis B infection  A7071: Chronic viral hepatitis B without delta-agent  EMISNQHO3: "EMISNQHO3"  A7070: Chronic viral hepatitis B with delta-agent  A7051: Acute delta-(super)infection of hepatitis B carrier | |  |

| Name | HCV_MA | |
| --- | --- | --- |
| Description | Born to mother with HCV | |
| Selection | Latest | |
| Referenced by sections |  | |
| Referenced by measures | Call08, Off08, Tst08, Ref08, Refe08 | |
| Read V2 Terms | | CTV3 Terms |
| 4JQD: Hepatitis C viral ribonucleic acid PCR positive  4JQF: Hepatitis C antigen positive  9NgR: On hepatitis C treatment plan  9kV: Hepatitis C screening positive - enhanced services admin  A70z0: Hepatitis C  EMISNQHE6: "EMISNQHE6"  ZV02C: [V]Hepatitis C carrier  A7072: Chronic viral hepatitis C  EMISNQHE11: "EMISNQHE11"  EMISNQHE29: "EMISNQHE29"  A70A: Hepatitis C genotype 1  A70G: Acute hepatitis C | |  |

| Name | CHILD_CARE | |
| --- | --- | --- |
| Description | Child in care | |
| Selection | Latest | |
| Referenced by sections |  | |
| Referenced by measures | Call09, Off09, Tst09, Ref09, Refe09 | |
| Read V2 Terms | | CTV3 Terms |
| 13IB0: Child in foster care  6A50: Child in care statutory review meeting  13Ii: Subject to care order under Children Act 1989  9Ngz9: In transition from children's to adult care service  13Ij: Subject to interim care order under Children Act 1989 | |  |

| Name | PRISON | |
| --- | --- | --- |
| Description | Prison | |
| Selection | Latest | |
| Referenced by sections |  | |
| Referenced by measures | Call10, Off10, Tst10, Ref10, Refe10 | |
| Read V2 Terms | | CTV3 Terms |
| 13HQ: In prison  13H9: Imprisonment record  ZV625: [V]Legal problems | |  |

| Name | ALTERED_ALT | |
| --- | --- | --- |
| Description | Altered ALT levels | |
| Selection | Latest | |
| Referenced by sections |  | |
| Referenced by measures | Call11, Off11, Tst11, Ref11, Refe11 | |
| Read V2 Terms | | CTV3 Terms |
| 44G2: Liver enzymes abnormal  44G31: ALT/SGPT level abnormal | |  |

| Name | HCV | |
| --- | --- | --- |
| Description | Hepatitis C | |
| Selection | Latest | |
| Referenced by sections |  | |
| Referenced by measures | Call12, Off12, Tst12, Ref12, Refe12 | |
| Read V2 Terms | | CTV3 Terms |
| 9kV: Hepatitis C screening positive - enhanced services admin  7Q053: RSV treatment and Hepatitis C treatment drugs Band 1  A7040: Viral hepatitis C with coma  A7050: Viral hepatitis C without mention of hepatic coma  A7072: Chronic viral hepatitis C  A70z0: Hepatitis C  ZV02C: [V]Hepatitis C carrier  Q409: Congenital viral hepatitis  ZV026: [V]Viral hepatitis carrier  14i: H/O hepatitis C antiviral drug therapy  EMISNQHE29: "EMISNQHE29" | |  |

| Name | HC_TEST | |
| --- | --- | --- |
| Description | Hepatitis C testing | |
| Selection | Latest | |
| Referenced by sections | HC_TEST, HC_TEST, HC_TEST | |
| Referenced by measures | Call12, Off12, Ref12, Refe12 | |
| Read V2 Terms | | CTV3 Terms |
| 2J1: Hepatitis C status  2J12: Hepatitis C non immune  43B7: Hepatitis C non-immune  43dD: Hepatitis C recombinant immunoblot assay  43h3: Hepatitis C PCR  43j5: Hepatitis C nucleic acid detection  43k1: Hepatitis C antigen level  43q: Hepatitis C virus RNA assay  43X2: Hepatitis C antibody test  43X3: Hepatitis C antibody test positive  43X6: Hepatitis C antibody level  4J3B: Hepatitis C viral load  65PM: Hepatitis C contact  65Q7: Viral hepatitis carrier  677Q: Hepatitis C screening counselling  6829: Hepatitis C screening  4JQC: Hepatitis C viral ribonucleic acid PCR negative  4JQE: Hepatitis C antigen negative  8I3v: Hepatitis C screening declined  677Q: Hepatitis C screening counselling  43j50: Hepatitis C nucleic acid detection assay  9kT: Hepatitis C screening negative - enhanced services admin  9kR: Chronic hepatitis annual review - enhanced services admin  EMISNQHE30: "EMISNQHE30"  ZV01B: [V]Contact with and exposure to viral hepatitis | |  |

| Name | HC_TEST_E | |
| --- | --- | --- |
| Description | Hepatitis C testing earliest | |
| Selection | Earliest | |
| Referenced by sections |  | |
| Referenced by measures | Tst12 | |
| Read V2 Terms | | CTV3 Terms |
| 2J1: Hepatitis C status  2J12: Hepatitis C non immune  43B7: Hepatitis C non-immune  43dD: Hepatitis C recombinant immunoblot assay  43h3: Hepatitis C PCR  43j5: Hepatitis C nucleic acid detection  43k1: Hepatitis C antigen level  43q: Hepatitis C virus RNA assay  43X2: Hepatitis C antibody test  43X3: Hepatitis C antibody test positive  43X6: Hepatitis C antibody level  4J3B: Hepatitis C viral load  65PM: Hepatitis C contact  65Q7: Viral hepatitis carrier  677Q: Hepatitis C screening counselling  6829: Hepatitis C screening  4JQC: Hepatitis C viral ribonucleic acid PCR negative  4JQE: Hepatitis C antigen negative  8I3v: Hepatitis C screening declined  677Q: Hepatitis C screening counselling  43j50: Hepatitis C nucleic acid detection assay  9kT: Hepatitis C screening negative - enhanced services admin  9kR: Chronic hepatitis annual review - enhanced services admin  EMISNQHE30: "EMISNQHE30"  ZV01B: [V]Contact with and exposure to viral hepatitis | |  |

| Name | REF | |
| --- | --- | --- |
| Description | Referred to Secondary Care Services | |
| Selection | Latest | |
| Referenced by sections | REF, REF, REF | |
| Referenced by measures |  | |
| Read V2 Terms | | CTV3 Terms |
| 8Hk5: Referred to hepatology service  EMISNQRE49: "EMISNQRE49" | |  |

| Name | X_TEST | |
| --- | --- | --- |
| Description | Patients to exclude from testing | |
| Selection | Latest | |
| Referenced by sections |  | |
| Referenced by measures |  | |
| Read V2 Terms | | CTV3 Terms |
| 2J11: Hepatitis C immune | |  |

| Name | OFFER | |
| --- | --- | --- |
| Description | Hep C screening offered | |
| Selection | Latest | |
| Referenced by sections | OFFER, OFFER | |
| Referenced by measures |  | |
| Read V2 Terms | | CTV3 Terms |
| 9Op1: Hepatitis C screening offered  6829: Hepatitis C screening | |  |

| Name | EOL | |
| --- | --- | --- |
| Description | Palliative care | |
| Selection | Latest | |
| Referenced by sections | EOL, EOL, EOL, EOL, EOL | |
| Referenced by measures |  | |
| Read V2 Terms | | CTV3 Terms |
| 1Z01: Terminal illness - late stage  2JE: Last days of life  8BA2: Terminal care  8BAP: Specialist palliative care  8BAS: Specialist palliative care treatment - daycare  8BAT: Specialist palliative care treatment - outpatient  8BAe: Anticipatory palliative care  8BJ1: Palliative treatment  8CM1%: On gold standards palliative care framework  !8CM15: (Excluding) GSF prognostic indicator stage A (blue) - yr plus prognosis  8CM4: Liverpool care pathway for the dying  8CME: Has end of life advance care plan  8H6A: Refer to terminal care consult  8H7L: Refer for terminal care  8H7g: Referral to palliative care service  8HH7: Referred to community specialist palliative care team  8IEE: Referral to community palliative care team declined  9EB5: DS 1500 Disability living allowance completed  9Ng7: On end of life care register  ZV57C: [V]Palliative care  8CMQ: On Liverpool care pathway for the dying  9NgD: Under care of palliative care service  9G8: Ambulance service notified of patient on EoL care register  9c0P: Current palliative oncology treatment  9c0N: Current supportive care for terminal illness  8CMW3: End of life care pathway  9K9: Palliative care handover form completed  9367: Patient held palliative care record  9c0L0: Planned palliative oncology treatment  9c0M: Planned supportive care for terminal illness  9NNd: Under care of palliative care specialist nurse  8CMb: Integrated care priorities for end of life  8CMg: End of life advance care plan  8B2a: Prescription of palliative care anticipatory medication  9NNf0: Under care of palliative care physician  38QH: Palliative Care Outcomes Collaboration Assessment Toolkit  38QK: Palliative Care Problem Severity Score | |  |

| Name | RIP | |
| --- | --- | --- |
| Description | Death | |
| Selection | Latest | |
| Referenced by sections |  | |
| Referenced by measures |  | |
| Read V2 Terms | | CTV3 Terms |
| 22J%: O/E - dead  9134: Registration ghost - deceased  94%: Death administration  !942: (Excluding) Medical cert. of still-birth  !94Z%: (Excluding) Death administration NOS  9234: FP22-death | |  |

| Name | ALT | |
| --- | --- | --- |
| Description | ALT levels | |
| Selection | Latest | |
| Referenced by sections |  | |
| Referenced by measures |  | |
| Read V2 Terms | | CTV3 Terms |
| 44G3%: ALT/SGPT serum level | |  |

| Name | XINVITE | |
| --- | --- | --- |
| Description | Exclude patient from invite column | |
| Selection | Latest | |
| Referenced by sections | XINVITE | |
| Referenced by measures |  | |
| Read V2 Terms | | CTV3 Terms |
| 682A: Hepatitis C screening not offered | |  |
